# Marker-based foot posture assessment in children

**DOI:** 10.1186/1757-1146-5-S1-O17

**Published:** 2012-04-10

**Authors:** Catriona M Kerr, Julie Stebbins, Tim Theologis, Amy B Zavatsky

**Affiliations:** 1 Department of Engineering Science, University of Oxford, Oxford, UK; 2 Oxford Gait Laboratory, Nuffield Orthopaedic Centre NHS Trust, Oxford, UK

## Background

An ideal measure of foot posture should be repeatable, representative of foot position, quantitative, objective, comprehensive, and suited to static and dynamic assessment. The Oxford Foot Model (OFM) is a clinically tested and validated model [1] used to assess foot deformity during walking. This study aims to use relevant components of the OFM to provide a quantitative foot posture assessment method. An assessment of OFM components which distinguish neutral, flat, and symptomatic flat feet is presented here.

## Materials and methods

A clinical assessment of the lower limbs was performed on 89 children (14 patients with symptomatic flat foot (SF, n=28 feet), and 75 volunteers with asymptomatic feet and no known pathology; 39 males, 50 females; 4.9 to 17.1 years old). Weightbearing clinical assessment of the asymptomatic group was used to classify the foot as normal (NN, n=81) or flat (NF, n=69). Reflective markers were placed at known locations on the lower limb and foot [1], and were tracked using a 12 camera Vicon MX system. Mean values of each OFM Euler angle were calculated during three seconds of quiet standing. Each foot was treated as an independent sample and ANOVA tests were used to assess whether OFM angles differed between groups.

## Results and discussion

**Figure 1:**
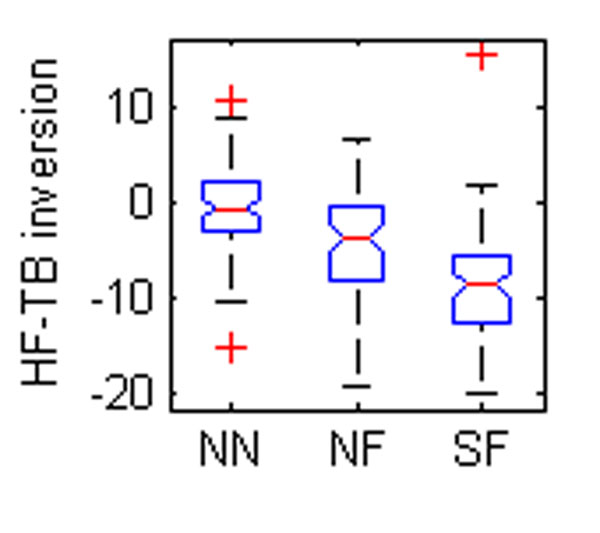
The box plots of mean angles of a standing trial found to be statistically different between groups. NN – normal child, neutral foot; NF – normal child, flat foot; SF – child with symptoms, flat foot. FF – forefoot, HF – hindfoot, TB – tibia

**Figure 2:**
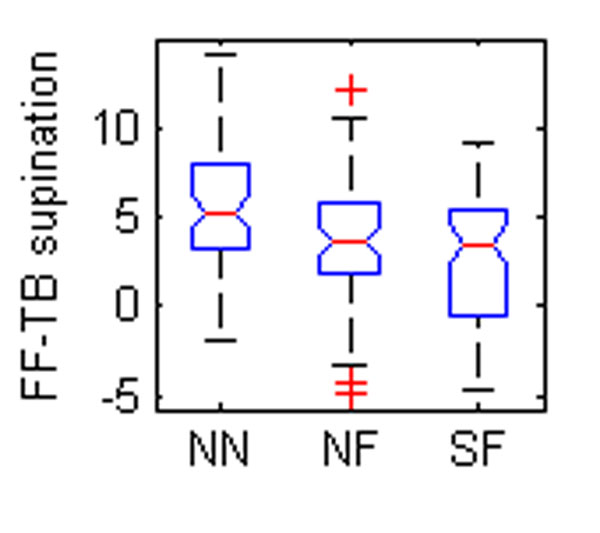
The box plots of mean angles of a standing trial found to be statistically different between groups. NN – normal child, neutral foot; NF – normal child, flat foot; SF – child with symptoms, flat foot. FF – forefoot, HF – hindfoot, TB – tibia

**Figure 3:**
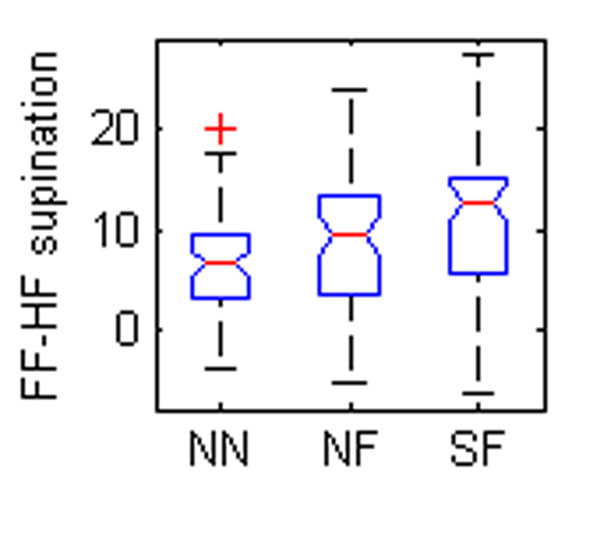
The box plots of mean angles of a standing trial found to be statistically different between groups. NN – normal child, neutral foot; NF – normal child, flat foot; SF – child with symptoms, flat foot. FF – forefoot, HF – hindfoot, TB – tibia

**Figure 4:**
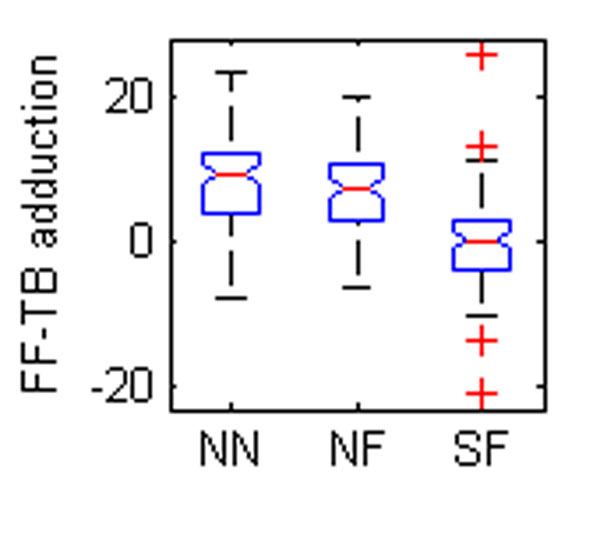
The box plots of mean angles of a standing trial found to be statistically different between groups. NN – normal child, neutral foot; NF – normal child, flat foot; SF – child with symptoms, flat foot. FF – forefoot, HF – hindfoot, TB – tibia

**Figure 5:**
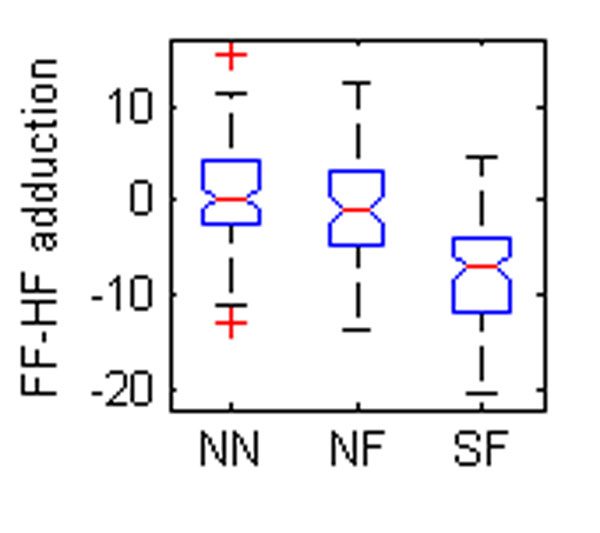
The box plots of mean angles of a standing trial found to be statistically different between groups. NN – normal child, neutral foot; NF – normal child, flat foot; SF – child with symptoms, flat foot. FF – forefoot, HF – hindfoot, TB – tibia

Five OFM angles were found to be different between groups (Table 1, Figure 1). The eversion of the hindfoot relative to the tibia was significantly different between all groups (Figure 1). Foot descriptions used for grouping are largely based on the degree of hindfoot eversion so a difference between normal and flat feet could be expected. The difference between SF and NF may reflect severity. The forefoot was also more pronated relative to the tibia in the flatfooted populations (Figure 2-3). This again could be a reflection of the original classification technique. The increased forefoot abduction relative to the hindfoot and tibia in the symptomatic population (Figure 4-5) may be a reflection of a midfoot break associated with more severe flat foot.

**Table 1:** p-values from ANOVA with Tukey post-hoc tests. Abbreviations described in Figure 1.

p-value	**HF–TB** dorsiflexion	**HF–T B** int. rotation	**HF–TB** inversion	**FF–TB** dorsiflexion	**FF–TB** adduction	**FF–TB** supination	**FF–HF** dorsiflexion	**FF– HF** adduction	**FF–HF** supination
**NN & NF**	0.999	0.581	*0.000	0.856	0.462	*0.013	0.529	0.069	0.118
**NN & SF**	0.999	0.719	*0.000	0.998	*0.000	*0.001	0.346	*0.000	*0.004
**NF & SF**	0.997	0.999	*0.004	0.944	*0.000	0.271	0.822	*0.000	0.171

## Conclusions

Elements of the OFM may be used to assess flat feet. Some measures have been shown to be associated only with symptomatic flat foot; these may be important in predicting the future for asymptomatic flat feet. The method is currently being applied to gait to determine if the parameters are relevant during walking.
